# Regulation of Pituitary MT1 Melatonin Receptor Expression by Gonadotrophin-Releasing Hormone (GnRH) and Early Growth Response Factor-1 (Egr-1): *In Vivo* and *In Vitro* Studies

**DOI:** 10.1371/journal.pone.0090056

**Published:** 2014-03-21

**Authors:** Sung-Eun Bae, Ian K. Wright, Cathy Wyse, Nathalie Samson-Desvignes, Pascale Le Blanc, Serge Laroche, David G. Hazlerigg, Jonathan D. Johnston

**Affiliations:** 1 Faculty of Health and Medical Science, University of Surrey, Guildford, Surrey, United Kingdom; 2 School of Biological Sciences, University of Aberdeen, Aberdeen, United Kingdom; 3 School of Veterinary Medicine, University of Glasgow, Glasgow, United Kingdom; 4 Centre de Neurosciences Paris-Sud, Centre National de la Recherche Scientifique, Univ. Paris-Sud, Orsay, France; 5 Department of Arctic and Marine Biology, Faculty of Biosciences Fisheries and Economy, University of Tromsø, Tromsø, Norway; University of Alabama at Birmingham, United States of America

## Abstract

Melatonin receptor expression exhibits profound developmental changes through poorly understood mechanisms. In mammals, a current model suggests that pubertal reactivation of gonadotrophin-releasing hormone (GnRH) secretion down-regulates MT1 melatonin receptors in pituitary gonadotroph cells, via the induction of early growth response factor-1 (EGR-1). Here we have examined this model by testing the hypotheses that inhibition of *Mt1* expression by GnRH occurs directly in gonadotroph cells, can be reversed in adulthood by blockade of GnRH receptors, and requires EGR-1. We first confirmed the endogenous expression of *Mt1* mRNA in the αT3-1 gonadotroph cell line. Stimulation of these cells with a GnRH agonist resulted in a rapid increase of *Egr-1* mRNA expression, which peaked after 30–60 minutes, and a more prolonged elevation of nuclear EGR-1 immunoreactivity. Moreover, the GnRH agonist significantly decreased *Mt1* mRNA. We then treated adult male rats with the GnRH antagonist cetrorelix or saline. After 4 weeks of daily injections, cetrorelix significantly reduced serum LH concentration and testis weight, with histological analysis confirming absence of spermatogenesis. Despite the successful inhibition of GnRH signalling, pituitary *Mt1* expression was unchanged. Next we studied the proximal region of the rat *Mt1* promoter. Consistent with previous work, over-expression of the transcription factor PITX-1 increased *Mt1*-luciferase reporter activity; this effect was dependent on the presence of consensus PITX-1 promoter binding regions. Over-expression of EGR-1 inhibited PITX-1-stimulated activity, even following mutation of the consensus EGR-1 binding site. Finally, we studied *Egr1* ^−/−^ mice and observed no difference in pituitary *Mt1* expression between *Egr1* ^−/−^ and wild-type litter mates. This work demonstrates that GnRH receptor activation directly down-regulates *Mt1* expression in gonadotroph cells. However, pituitary *Mt1* expression in adults is unaltered by blockade of GnRH signalling or absence of EGR-1. Our data therefore suggest that melatonin receptor regulation by GnRH is not reversible in adulthood and doesn't require EGR-1.

## Introduction

The hormone melatonin is implicated in multiple diverse aspects of physiology [1]. It is secreted into the blood and cerebrospinal fluid by the pineal gland, and is produced locally by other tissues within the body, such as the retina [2]. In mammals, melatonin signals through two receptors of the G-protein-coupled super-family, termed MT1 and MT2 [3]. Compared to adults, foetuses and neonates exhibit a more widespread receptor distribution, suggesting that melatonin may have as yet unknown roles in development [4]. Surprisingly little is known about the mechanisms controlling these developmental changes in melatonin signalling.

Pineal melatonin production is driven by the master circadian clock in the suprachiasmatic nuclei of the hypothalamus and thus exhibits a robust daily rhythm. This rhythm varies in proportion to the length of the night and so melatonin encodes both daily and seasonal time [5]. In mammals, melatonin is essential for photoperiodic physiology and can regulate circadian clock gene expression in several peripheral tissues [6]–[9], indicating a possible ability to synchronise peripheral circadian clocks. In addition to control of rhythmic physiology, melatonin is also reported to control many other biological processes. One of these is suppression of the endocrine response of the developing pituitary gland to the key reproductive factor, gonadotrophin-releasing hormone (GnRH) [10]. This effect disappears in the postnatal rodent pituitary gland and thus may be relevant to the timing of puberty [11]–[13]. Interestingly, melatonin secretion has been associated with reproductive development and the timing of human puberty in some studies [14]–[16]. However aspects of this work has methodological flaws [14], [15] and other studies have failed to replicate the finding [17], [18].

We have previously studied the regulation of MT1 melatonin receptors in the pituitary gland and suggested a mechanism controlling MT1 expression during reproductive development. In our model, *Mt1* promoter activity is stimulated by the transcription factor pituitary homeobox-1 (PITX-1) [19]–[21]. During early stages of development, PITX-1-stimulation of *Mt1* is thought to be inhibited by factors involved in Rathke's Pouch proliferation, such as MSX-1 [22]. Consistent with this hypothesis, the decline in *Msx-1* coincides with the onset of *Mt1* expression in the foetal rat pituitary. Following a period of melatonin sensitivity, it is proposed that the pubertal reactivation of GnRH secretion then finally down-regulates *Mt1* expression, likely via induction of early growth response factor-1 (EGR-1; also known as NGFI-A and Krox-24) [20], [21].

This model received preliminary support from the observation that adult *hypogonadal* mice, which are unable to synthesise GnRH, exhibit elevated levels of *Mt1* expression than their wild type controls [20]. However, the model is yet to be thoroughly tested. In particular, it is unclear whether GnRH directly regulates gonadotroph MT1 expression, whether the inhibitory effects of GnRH require EGR-1 and are reversible in adulthood. Here, we have addressed these questions using a combination of in vivo and in vitro techniques. As in previous work by ourselves and others, much of the data derives from the rat, in which developmental changes of *Mt1* are most extensively characterised. Due to the availability of suitable gonadotroph cell lines and transgenic ‘knockout’ animals, other parts of the study have used mouse tissue. Such an approach takes advantage of the benefits of each system and has been used successfully before, e.g. [20].

## Methods

### Cell culture and transient transfection assays

Unless otherwise specified, all cells were cultured at 37°C and 5% CO_2_ in growth medium: DMEM (Invitrogen Ltd, Paisley, UK) supplemented with 10% fetal bovine serum (Invitrogen), antibiotic/antimycotic (Invitrogen), and sodium pyruvate (Sigma-Aldrich Co Ltd, Poole, UK). Data shown are from a representative of at least three independent experiments.

For studies of GnRH signalling, αT3-1 cells [23] were seeded in 6 well plates at a density of 300,000 cells per well. After 24 hours, cells were treated with GnRH agonist ([des-Gly^10^_,_ D-ala^6^ ]-LH-RH ethylamide acetate salt hydrate; Sigma-Aldrich) at final concentration of 100 nM. After the required treatment time(s), cells were harvested for analysis of mRNA by TaqMan real-time PCR or protein by western blot, as described below.

For studies of rat *Mt1* promoter activity, COS-7 cells (ATCC; LGC Standards, Teddington UK) were seeded in 96 well plates at a density of 3,500 cells per well. After 24 hours, cells were transfected using FuGene6 reagent (Roche Diagnostics Ltd, Burgess Hill, UK), according to the manufacturer's protocol. Each well received DNA containing 5 ng of MT1-luciferase reporter plasmid, and appropriate expression vectors, made up to a total of 50 ng with pcDNA3. Forty-eight hours after transfection, reporter gene activity was measured using the Dual-Glo system (Promega UK, Southampton, UK). Each treatment was performed in quadruplicate wells per experiment. Rat *Mt1*-luciferase plasmids were based on the −445 bp vector described previously [20]. Additional plasmids were manufactured by Eurofins MWG Operon (Ebersberg, Germany) to include mutation in the EGR-1 or one of the two PITX-1 binding sites described previously [19], [20]. The distal PITX-1 site (P1) was modified from TCATCC to TGGCGC; the proximal PITX-1 site (P2) was modified from TAATCC to TGGCGC; the EGR-1 site was modified from AGGCGCGGGAGG to AGGCTCTTTAGG.

### Ethics Statement

Experiments using rats were performed in accordance with the UK Animals (Scientific Procedures) Act, 1986, under licence from the UK Home Office (PPL 70/7059). Experiments were also approved by the University of Surrey's Animal Welfare Ethical Review Board. All experimental work with mice was conducted in accordance with the European Communities Council Directive 86/609/EEC and the French National Committee (87/848). No surgical procedures were undertaken in this study. Animal suffering was minimised by sacrificing animals according to approved procedures (rising concentration of CO_2_, cervical dislocation).

### Animals

Twelve 10-week old male Wistar rats were obtained from Charles River UK. After acclimation to the experimental facility, rats were treated for 4 weeks with daily i.p. injections of either 100 µg GnRH antagonist (cetrorelix acetate; Merck Serono, Feltham, UK) or saline control (n = 6 animals per group, based on in situ hybridisation data comparing hypogonadal and wild type adult mice [20]). Injections were given between 10:00–11:00 each day. Experimental groups were weight-matched and individual animals housed in separate cages under a 12-hour light: 12-hour dark cycle with ad libitum access to food and water. Following the treatment period, rat brains and pituitaries were dissected together, keeping the pituitary stalks intact, and frozen on dry ice prior to analysis by in situ hybridisation histochemistry. Serum samples were collected for luteinising hormone (LH) analysis. Both testes from each animal were weighed and frozen on dry ice prior to histological analysis. All samples were stored at −80°C.

Egr-1^−/−^ mice and wild type littermates were bred in an established colony at Université Paris-Sud, described elsewhere [24]. Brains and pituitaries from adult mice were dissected together, keeping the pituitary stalks intact, and frozen on dry ice prior to analysis by in situ hybridisation histochemistry. The number of animals used for analysis (n = 7 per genotype) was based on in situ hybridisation data comparing hypogonadal and wild type adult mice [20].

### Serum LH analysis

Serum LH was measured using a rodent LH ELISA kit (Endocrine Technologies, INC. Newark, CA, USA), according to the manufacturer's instructions. In brief, 50 µl of sample or standard was mixed with 100 µl of enzyme conjugate and incubated at 37°C for 2 hours. Assay plate wells were rinsed before 100 µl of TMB solution was added and incubated at room temperature for 20 mins, in the dark. Finally the reaction was stopped by adding 50 µl of 2N HCl and the optical density was measured at 450 nm using a microtiter well reader. Concentration of LH was calculated from the standard curve.

### Testis histology

Sections of frozen testis (7 µm) were prepared and post-fixed with ice-cold 4% paraformaldehyde for 10 mins then processed for hematoxylin and eosin staining. Sections were examined for general morphology using light microscopy.

### *In situ* hybridisation histochemistry

Analysis of *Mt1* mRNA expression in brain/pituitary sections was performed using a well validated assay, as described previously [20]. In brief, 20 µm sagittal sections of brain and pituitary tissue were hybridised with a ^35^S-labelled riboprobe corresponding to nucleotides 30–466 of GenBank accession number U14409. Hybridisation signal was quantified against optical density standards on each autoradiography film.

### TaqMan real-time PCR

After treatment, cells were washed twice with warmed 1× PBS. Total RNA extraction and cDNA synthesis were performed as described previously [25]. Expression of mRNA for *Mt1*, *Egr-1* and *Gapdh* was measured using TaqMan RT-PCR [25]. *Mt1* forward primer: 5′-TCTGCTACGTGTTCCTGATATGGAT-3′; *Mt1* reverse primer: 5′-TGGAGTGTTCCGGTTTGCA-3′; *Mt1* probe: 5′(FAM)-CTGACACTCATCGCCATCATGCCC-3′(TAM); *Egr-1*forward primer: 5′-CCTTTTCTGACATCGCTCTGAA-3′; *Egr-1* reverse primer: 5′-GGCAACCGAGTCGTTTGG-3′; *Egr-1* probe: 5′(FAM)-CTCGTCTCCACCATCGCCTTCTCATT-3′(TAM).

### Western blots

Cytoplasmic and nuclear-enriched protein was extracted from cells using the NE-PER kit (Thermo Scientific, Cramlington, UK). From each sample, 30 µg of total protein was separated on a 10% polyacrylamide gel. Protein was transferred from gels to methanol-activated PVDF membranes, which were then incubated in wash buffer (TRIS-buffered saline containing 0.1% TWEEN 20; 3×5 minutes at room temperature), blocking buffer (5% skimmed milk powder in wash buffer; 60 minutes at room temperature), and wash buffer (3×5 minutes at room temperature).

Membranes were incubated with 1∶200 dilution of anti-EGR-1 antibody (Egr-1 (588); Santa Cruz Biotechnology Inc, Heidelberg, Germany) in blocking buffer for 60 minutes at room temperature. After rinsing in wash buffer (3×5 minutes, room temperature), membranes were then incubated with a 1∶5000 dilution of horseradish peroxidase-coupled anti-rabbit secondary antibody (Sigma-Aldrich) in blocking buffer for 60 minutes at room temperature. Next, membranes were rinsed in wash buffer (1×5 minutes, room temperature) and protein expression detected using the enhanced chemiluminescence (ECL; GE Healthcare, UK) system according to the manufacturer's protocol.

Following detection of EGR-1 protein, membranes were briefly rinsed in ddH2O and wash buffer, before being incubated in strip buffer (25 mM glycine, 1% SDS; 25 minutes, room temperature) and wash buffer (2×5 minutes, room temperature). Non-specific binding was blocked as described above and membranes were then incubated with 1∶2000 dilution of anti-actin antibody (Sigma-Aldrich) in blocking buffer for 60 minutes at room temperature. Washing, secondary antibody incubation and ECL detection were then performed as described above.

### Statistics

Quantitative data are presented as mean ± SEM and were analysed by either unpaired t-test or one-way ANOVA with Bonferroni post-hoc test, as appropriate. Units of analysis were data from either one animal or one well of cells. Statistical significance was defined as p<0.05.

## Results

### Regulation of *Egr-1* and *Mt1* by GnRH agonist treatment of αT3-1 gonadotroph cells

**Figure 1:**
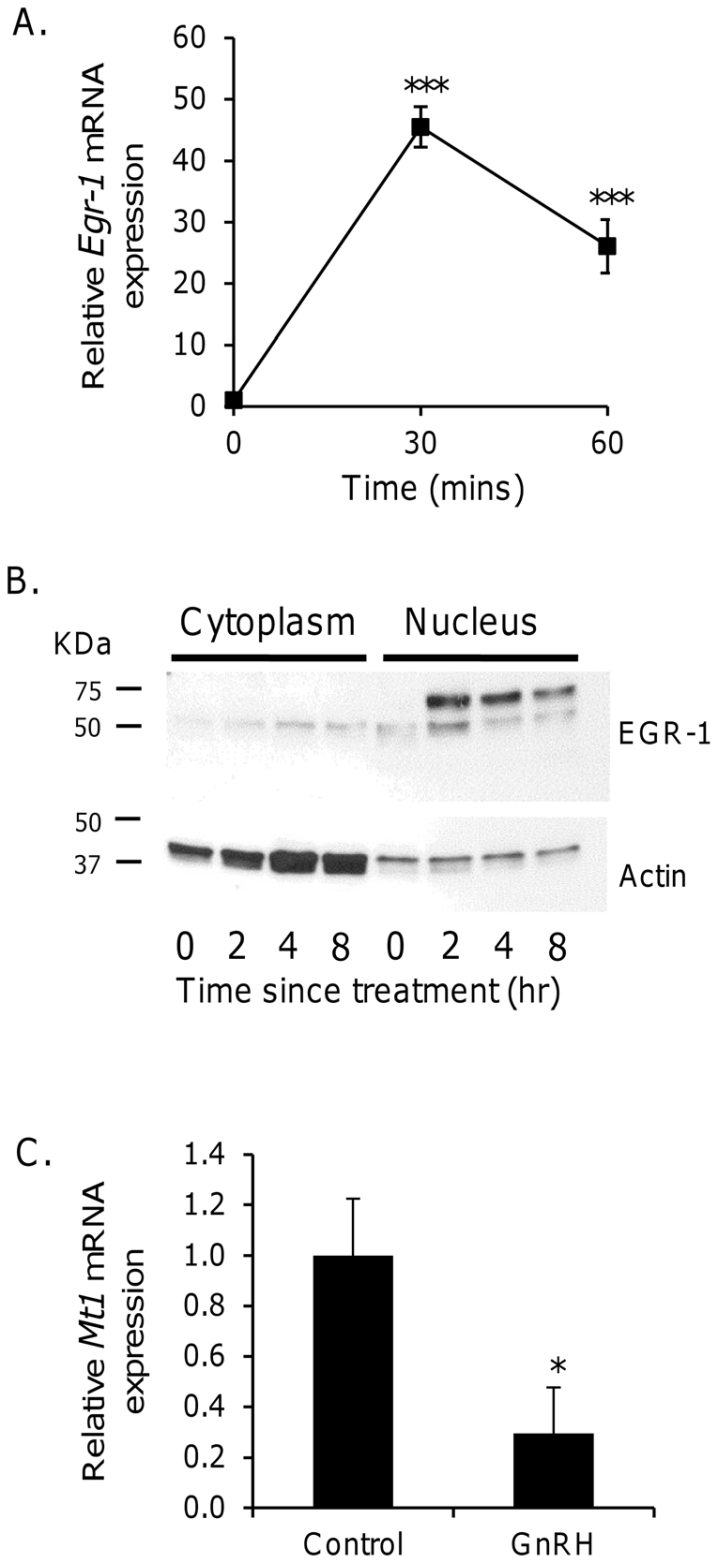
GnRH agonist treatment induces EGR-1 and inhibits *Mt1* expression in αT3-1 gonadotroph cells. Cells were treated with 100(A) Total mRNA was extracted for analysis of *Egr-1* mRNA, expressed relative to *Gapdh*, by qRT-PCR. *** p<0.001 vs time 0 (one-way ANOVA with Bonferroni post-hoc test). (B) Cytoplasmic and nuclear-enriched lysates were prepared for analysis of EGR-1 and actin protein expression by western blot. (C) Following 20 hours of GnRH agonist treatment, total mRNA was extracted for analysis of *Mt1* mRNA, expressed relative to *Gapdh*, by qRT-PCR. * p<0.05 control vs treated group (unpaired t-test).

Treatment of αT3-1 cells with GnRH agonist induced a significant (p<0.001, one-way ANOVA) induction of *Egr-1* mRNA expression (Fig. 1A). Maximal expression was observed after stimulation for 30 minutes, with a partial decline apparent 30 minutes later. In unstimulated cells, there was a faint band of EGR-1 immunoreactivity at approximately 50 kDa in both cytoplasmic and nuclear-enriched samples (Fig. 1B). Following stimulation, there was little change in cytoplasmic EGR-1 expression; however, analysis of nuclear protein revealed increased expression of the 50 kDa band at 2 hours, with strong expression of an approximately 65 kDa band of immunoreactivity between 2–8 hours. Finally, 20 hours after onset of GnRH agonist treatment, there was a significant (p<0.05, unpaired t-test) decrease in *Mt1* mRNA expression (Fig. 1C). No significant decline of *Mt1* mRNA expression was observed at earlier time points (data not shown).

### Treatment of rats with GnRH receptor antagonist

**Figure 2:**
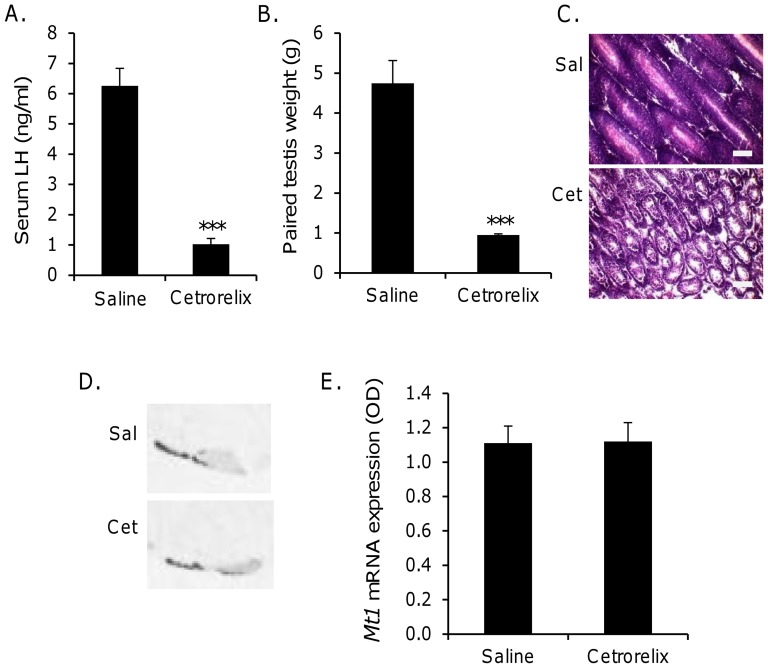
Treatment of rats with GnRH antagonist impairs reproductive status but does not affect *Mt1* expression. Male Wistar rats were given a daily i.p. injection of the GnRH antagonist cetrorelix (100 µg/day) or saline control for 4 weeks. (A) Serum LH was measured by ELISA. (B–C) Testis morphology was assessed by (B) paired testis weight and (C) histological analysis of tissue sections using hematoxylin and eosin staining. Scale bar = 25 µm (D–E) Brain and pituitary tissue from saline and cetrorelix-treated rats was dissected with the pituitary stalk intact, frozen on dry ice and stored at −80°C. Twenty micron sagittal sections were cut and *Mt1* mRNA expression determined by in situ hybridisation histochemistry. (D) Representative autoradiographs. In both treatment groups, strong pituitary expression was observed in the pars tuberalis and along the rostral extent of the ventral pars distalis; weaker expression was observed throughout the rest of the pars distalis. (E) Quantification of *Mt1* expression by densitometry. *** p<0.001 saline vs cetrorelix group (unpaired t-test). Sal: saline-treated; Cet: cetrorelix-treated.

Daily injection of rats with cetrorelix impaired reproductive function, as revealed by a significant (p<0.001, unpaired t-test) reduction of both serum LH concentration (saline: 6.26±0.58 ng/ml; cetrorelix: 1.03±0.18 ng/ml; Fig. 2A) and paired testis weight (4.75±0.57 g; cetrorelix: 0.95±0.03 g; Fig. 2B). On histological analysis, all testes from saline-treated rats exhibited seminiferous tubules full of developing spermatozoa, whereas testes from cetrorelix-treated individuals exhibited smaller seminiferous tubules in which there was no evidence of spermatogenesis (Fig. 2C).

Expression of *Mt1* mRNA was analysed by in situ hybridisation of sagittal sections through brain and pituitary tissue. In both treatment groups, strong pituitary expression was observed in the pars tuberalis and along the rostral extent of the ventral pars distalis; weaker expression was observed throughout the rest of the pars distalis (Fig. 2D). Quantification of pituitary *Mt1* expression revealed no significant difference (p>0.05, unpaired t-test) in densitometry between the two treatment groups (saline: 1.11±0.10 OD units; cetrorelix: 1.12±0.11 OD units; Fig. 2E).

### Molecular analysis of rat *Mt1* promoter activity *in vitro*

**Figure 3:**
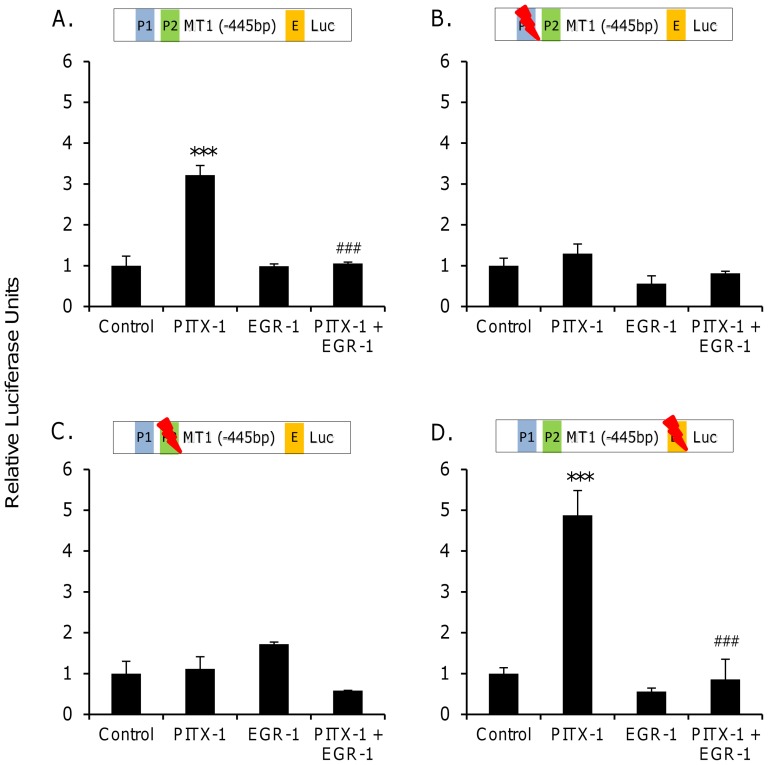
Regulation of rat *Mt1* promoter activity by PITX-1 and EGR-1 in vitro. COS-7 cells were co-transfected with an *Mt1*-luciferase reporter construct and a combination of control vector, PITX-1 expression vector and EGR-1 expression vector. Horizontal bars indicate mutagenesis of PITX-1 and EGR-1 binding sites. P1: distal PITX-1 consensus site; P2: proximal PITX-1 consensus site; E: EGR-1 consensus site. *** p<0.001 vs control group; ### p<0.001 vs PITX-1 group (one-way ANOVA with Bonferroni post-hoc test).

Activity of the unmodified *Mt1* promoter was significantly (p<0.001, one-way ANOVA) modified by experimental conditions (Fig. 3A), such that co-transfection with PITX-1 expression vector alone significantly (p<0.001, Bonferroni post-hoc test) increased promoter activity compared to the control group. Mutation of either of the PITX-1 consensus sequences abolished the ability of PITX-1 to stimulate the *Mt1* promoter, as there was no significant (p>0.05) difference in promoter activity between control and PITX-1-stimulated groups (Fig. 3B and 3C). Following mutation of the EGR-1 consensus sequence, there was again a significant (p<0.001, one-way ANOVA) effect of co-transfection conditions on *Mt1* promoter activity; specifically, PITX-1 stimulated the *Mt1* promoter and EGR-1 remained able to inhibit PITX-1-stimulated activity (p<0.001, Bonferroni post-hoc test; Fig. 3D).

### Expression of *Mt1* mRNA in the pituitary of *Egr-1^−/−^* mice

**Figure 4:**
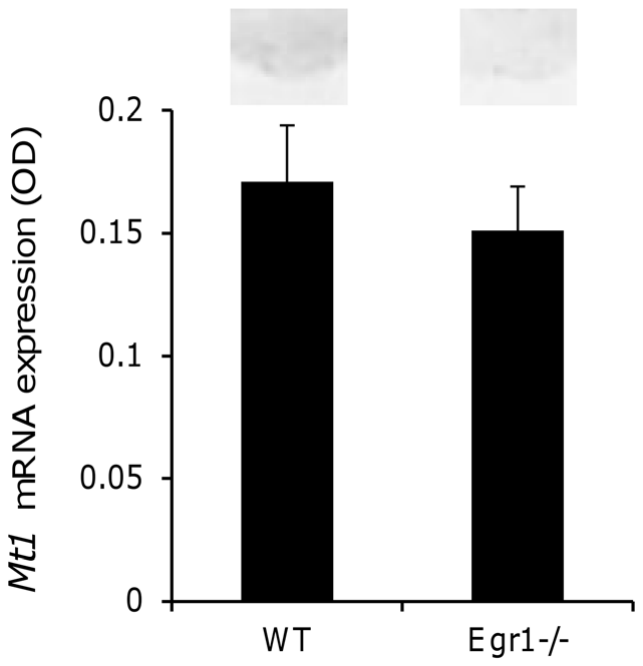
Pituitary *Mt1* expression is unaltered in adult *Egr-1^−^* ^/−^ mice. Brain and pituitary tissue from adult wild type (WT) and *Egr-1^−^* ^/−^ mice was dissected with the pituitary stalk intact, frozen on dry ice and stored at −80°C. Twenty micron sagittal sections were cut and *Mt1* mRNA expression determined by in situ hybridisation histochemistry. Quantification of *Mt1* expression by densitometry revealed no significant difference (p>0.05 unpaired t-test) of genotype. Representative autoradiographs are shown above the respective bar.

Sagittal sections of brain and pituitary tissue from *Egr-1^−/−^* mice and wild type litter mates were analysed by in situ hybridisation. In mice of both genotypes, faint *Mt1* expression was observed in the pituitary pars tuberalis region. However, quantification revealed no significant difference (p>0.05, unpaired t-test) in densitometry between the two genotypes (wild type: 0.17±0.02 OD units; Egr1^−/−^: 0.15±0.02 OD units; Fig. 4).

## Discussion

This study demonstrates that activation of GnRH receptors in gonadotroph cells down-regulates expression of *Mt1* mRNA. Despite this, functional blockade of GnRH receptors in adult rats for 4 weeks fails to alter in vivo expression of *Mt1*. In transient transfection assays, over-expression of EGR-1 inhibits PITX-1-stimulated rat *Mt1* promoter activity independently of an EGR-1 consensus sequence. However, there is no difference in pituitary *Mt1* expression in *Egr-1* ^−/−^ mice and wild type controls.

Our previous studies led us to hypothesise that the perinatal decline in pituitary MT1 melatonin receptor expression is due to the pubertal reactivation of GnRH secretion from the hypothalamus. We therefore first studied *Mt1* expression in murine αT3-1 gonadotroph cells, which model newly differentiated gonadotrophs as they express the common glycoprotein alpha subunit (αGSU) and functional GnRH receptors, but not the LH beta subunit [23], [26]. Here, we demonstrate that αT3-1 cells also express *Mt1* mRNA, making them an ideal model to study the interaction between GnRH and endogenous melatonin receptors. As described previously, stimulation of αT3-1 cells with a GnRH agonist rapidly induces transient expression of *Egr-1* mRNA [27], with a more prolonged induction of EGR-1 protein in nuclear-enriched extracts. Following this induction of nuclear EGR-1 protein, we observed a significant decrease in *Mt1* mRNA. Allowing for a delay between *Mt1* transcriptional inhibition and decrease in steady state mRNA levels, the relative time course of these events may be consistent with a functional relationship between EGR-1 and *Mt1* in perinatal gonadotroph cells. The half life of *Mt1* mRNA is estimated to be 2–3 hours in ovine pars tuberalis cells [28]. Despite differences in cell type and unknown extent of transcriptional repression in our GnRH-treated αT3-1 cells, the timing of *Mt1* inhibition is not inconsistent with its estimated half life. However, attempts to demonstrate a causal relationship between these events were prevented by an inability to transfect the αT3-1 cells with inhibitors of EGR-1 expression or function.

Our previous in vivo data demonstrated that adult rodents unable to synthesise GnRH throughout development exhibit elevated pituitary *Mt1* expression [20], but the regulation of *Mt1* by GnRH signalling in adulthood is unknown. We therefore next investigated the effect of a GnRH receptor antagonist, cetrorelix, on *Mt1* expression in the adult rat pituitary. Daily intra-peritoneal injections of cetrorelix successfully shut-down the rats' reproductive system, as demonstrated by analysis of serum LH concentration and testis morphology. However, despite this physiological effect, there was surprisingly no change in pituitary *Mt1* expression. This finding contrasts with the ability of cetrorelix to induce MT1 receptor expression in the GT1-7 neuronal cell line [29] and suggests that changes during normal gonadotroph development maintain inhibition of *Mt1* mRNA, despite the lack of GnRH signalling.

A limitation of the current study is that our in situ hybridisation protocol measured gene expression in all cell types present in the tissue sections and not just gonadotroph cells. However, to explain our cetrorelix data, any elevation of gonodotroph *Mt1* mRNA caused by the treatment would have to be mirrored by an equal decrease in *Mt1* expression within other cell types. Moreover, the increased *Mt1* mRNA observed in *hypogonadal* mice was readily detectable by the same in situ hybridisation protocol [20]. The most likely explanation of our results is therefore that cetrorelix had no effect on gonadotroph *Mt1* expression in the adult rat pituitary. It also remains possible that adult mice treated with cetrorelix may exhibit a similar increase in pituitary *Mt1* mRNA expression as we previously observed in *hypogonadal* mice. However the species-specific mechanisms that could cause such a difference are unclear.

We next extended previous analyses of rat *Mt1* promoter activity in vitro. As shown previously [20], over-expression of PITX-1 induces activity of a −445 bp *Mt1*-luciferase construct and this PITX-1-stimulated activity is strongly inhibited by co-transfection with an EGR-1 expression vector. The ability of PITX-1 to stimulate *Mt1* promoter activity was inhibited by mutagenesis of either of its consensus sequences, indicating that both are required for successful promoter activation. However, EGR-1 retained its ability to inhibit PITX-1-stimulated promoter activity even after mutation of its consensus binding sequence. This finding suggested that, in our in vitro system, EGR-1 is able to inhibit *Mt1* promoter activity without binding to DNA and thus presumably via protein-protein interactions. Such a mechanism would be consistent with reports of functional interactions between EGR-1 and other proteins involved in transcriptional regulation [27], [30]–[33].

Finally, in order to investigate the role of EGR-1 in melatonin receptor regulation in vivo, we examined *Mt1* expression in the pituitary of *Egr-1* ^−/−^ mice. As observed previously [20], adult wild type mice exhibited weak pituitary *Mt1* expression. In contrast to the upregulation of *Mt1* in *hypogonadal* mice that are unable to synthesise GnRH, and despite inhibition of *Mt1* promoter activity by EGR-1 in vitro, there was no difference in pituitary *Mt1* expression between *Egr-1* ^−/−^ mice and wild type litter mates. Thus, despite the ability of EGR-1 over-expression to inhibit *Mt1* promoter activity in vitro, EGR-1 is not necessary for GnRH to regulate *Mt1* in vivo. One possible explanation for this finding is that there is developmental compensation in the knock-out model [34]. However, *Egr-1* ^−/−^ mice remain infertile due to a lack of LH synthesis [35], [36], indicating that developmental compensation within the pituitary would have to be specific for *Mt1* regulation. A second and perhaps more likely explanation for the absence of an effect of genotype is that additional pathway(s) link GnRH signalling to *Mt1* expression, thus providing functional redundancy of signal transduction mechanisms. At present we are unable to distinguish between these possibilities.

In summary, we have provided novel information describing the regulation of pituitary *Mt1* melatonin receptor mRNA, both in vivo and in vitro. Although underlying signal transduction mechanisms are unclear, our current data expand upon previous work and reveal a direct ability of GnRH signalling to down-regulate melatonin receptor expression in αT3-1 gonadotroph cells. Despite this effect and the elevated *Mt1* previously observed in *hypogonadal* mice, blockade of GnRH signalling in adult animals didn't increase pituitary *Mt1* mRNA. Together these findings suggest that GnRH may induce a long-term change in melatonin sensitivity, rather than merely a tonic inhibition of gene transcription. As discussed elsewhere [21], the developmental timing of pituitary melatonin receptor down-regulation is similar to the onset of hypothalamic GnRH signalling in rodents and sheep, which have greatly differing gestational lengths. Moreover, rat and ovine *Mt1* promoters possess some common regulatory elements [20], [21]. The findings presented herein may therefore be relevant to multiple mammalian species.
